# Prediction of early hepatocellular carcinoma recurrence using germinal center kinase-like kinase

**DOI:** 10.18632/oncotarget.10176

**Published:** 2016-06-20

**Authors:** Cheng-Hsun Ho, Huai-Chia Chuang, I-Chin Wu, Hung-Wen Tsai, Yih-Jyh Lin, Hung-Yu Sun, Kung-Chia Young, Yen-Cheng Chiu, Pin-Nan Cheng, Wen-Chun Liu, Tse-Hua Tan, Ting-Tsung Chang

**Affiliations:** ^1^ Research Center of Clinical Medicine, National Cheng Kung University Hospital, College of Medicine, National Cheng Kung University, Tainan, Taiwan; ^2^ Department of Internal Medicine, National Cheng Kung University Hospital, College of Medicine, National Cheng Kung University, Tainan, Taiwan; ^3^ Immunology Research Center, National Health Research Institutes, Zhunan, Taiwan; ^4^ Department of Pathology, National Cheng Kung University Hospital, College of Medicine, National Cheng Kung University, Tainan, Taiwan; ^5^ Infectious Disease and Signaling Research Center, National Cheng Kung University, Tainan, Taiwan; ^6^ Department of Surgery, National Cheng Kung University Hospital, College of Medicine, National Cheng Kung University, Tainan, Taiwan; ^7^ Department of Medical Laboratory Science and Biotechnology, College of Medicine, National Cheng Kung University, Tainan, Taiwan; ^8^ Department of Pathology & Immunology, Baylor College of Medicine, Houston, Texas, USA; ^9^ Institute of Molecular Medicine, College of Medicine, National Cheng Kung University, Tainan, Taiwan

**Keywords:** hepatocellular carcinoma, recurrence, GLK, NFκB

## Abstract

Germinal center kinase-like kinase (GLK) is a key controller of autoimmunity. In this study, we assessed the clinical relevance and tumorigenic effects of GLK in hepatocellular carcinoma (HCC). Using immunohistochemistry, we showed that the GLK proportion score increased in both cancerous and adjacent non-cancerous liver tissue from patients with HCC recurrence. A Kaplan-Meier analysis revealed that patients with a wide distribution of GLK in non-cancerous liver tissue had a higher rate of HCC recurrence than those with very low or no GLK expression. Multivariate Cox regression analyses indicated that a high GLK proportion score in non-cancerous liver tissue was an independent predictor of early HCC recurrence after resection. Lentiviral vector-mediated overexpression of GLK activated the nuclear factor kappa B (NFκB) signaling cascade and accelerated cell cycle progression in primary human hepatocytes, thereby promoting proliferation. An increase in GLK expression coincided with NFκB activation and enhanced expression of proliferating cell nuclear antigen in HCC tissue. Our findings demonstrate a potential hepatocarcinogenic effect of GLK and the feasibility of using GLK to predict early HCC recurrence.

## INTRODUCTION

Hepatocellular carcinoma (HCC) is the third most common cause of cancer-related deaths globally [1]. There are approximately three quarters of a million new cases of HCC and 700,000 deaths from HCC each year. Risk factors for HCC include chronic hepatitis B virus (HBV) or hepatitis C virus (HCV) infection, alcoholic liver disease, and non-alcoholic fatty liver disease [2, 3]. Advances in imaging techniques for early detection, surgical methods, and the development of novel therapies have drastically improved the prognosis of patients with HCC and minimized complications [4, 5]. However, the high rate of recurrence following hepatic resection substantially reduces the overall survival of patients. The average five-year cumulative rate of HCC recurrence is greater than 70% globally [6, 7]. Relapse is the major cause of death of HCC patients but the factors that drive HCC recurrence are incompletely understood. Therefore, it is imperative to identify a marker for predicting early recurrence and patient prognosis, and for facilitating the optimal clinical management of patients following hepatectomy.

Germinal center kinase-like kinase (GLK/MAP4K3), a member of the mitogen-activated protein kinase kinase kinase kinase (MAP4K) family of proteins, is a Ste20-like serine-threonine kinase [8]. GLK has a conserved, N-terminal kinase domain, a conserved C-terminal citron homology domain, and multiple proline-rich motifs in the central region of the protein. It modulates the mammalian target of rapamycin (mTOR) complex 1 after treatment with amino acids [9, 10] and determines cell death through post-transcriptional regulation of BH3-only proteins [11]. GLK also stimulates the protein kinase C (PKC)-θ-dependent nuclear factor kappa B (NFκB) signaling pathway in T cells, and this activity is directly correlated with the severity of systemic lupus erythematosus [12]. GLK regulates various cellular processes, however its role in cancer development and recurrence is unclear. In this study, we investigated the clinical relevance of GLK in early HCC recurrence, and elucidated the effects of GLK activity on hepatocyte proliferation.

## RESULTS

### Patient characteristics

The demographic and clinical data for 69 patients with HCC are shown in Table 1. There was a 3:1 ratio of men to women. Approximately one-fifth of the patients were dependent on alcohol or smoking. Thirty percent of the patients had been diagnosed with fatty liver and 38% with liver cirrhosis. More than 50% of the patients had HBV and more than 30% had HCV. Histology data indicated that patients had slight hepatic necroinflammation and moderate fibrosis. Patients had increased serum alanine aminotransferase, aspartate aminotransferase, and α-fetoprotein levels, but normal albumin, total bilirubin, and prothrombin times. Based on the TNM classification of malignant tumors, 22 patients were categorized as stage one, 36 as stage two, and 11 as stage three. Thirty-two patients were diagnosed with HCC recurrence during post-hepatectomy follow-up. These patients had lower albumin levels (*P* = 0.002), advanced-stage tumors (*P* < 0.001), and higher mortality rates (*P* = 0.044) than those without recurrence (Table 1).

**Table 1:** Characteristics of patients with hepatocellular carcinoma (n = 69)

Variable	Total (n = 69)	Recurrence (n = 32)	Non-recurrence (n = 37)	*P*-value
Sex (M:F)	51:18	24:8	27:10	1.000
Age (years)	60.2 ± 10.9	60.3 ± 8.5	60.2 ± 12.7	0.971
Body mass index	24.6 ± 3.4	24.5 ± 4.1	24.7 ± 2.8	0.785
Alcohol	18.8% (13/69)	15.6% (5/32)	21.6% (8/37)	0.556
Smoking	21.7% (15/69)	15.6% (5/32)	27.0% (10/37)	0.381
Fatty liver	30.4% (21/69)	18.8% (6/32)	40.5% (15/37)	0.068
HBsAg (+)	55.1% (38/69)	59.4% (19/32)	51.4% (19/37)	0.628
HCV RNA (+)	27.5% (19/69)	37.5% (12/32)	24.3% (9/37)	0.298
HBsAg (+) and HCV RNA (+)	2.9% (2/69)	3.1% (1/32)	2.7% (1/37)	1.000
Knodell inflammation score	3.7 ± 2.0	4.1 ± 1.7	3.4 ± 2.2	0.131
Ishak fibrosis score	4.0 ± 1.7	4.4 ± 1.7	3.6 ± 1.6	0.056
Liver cirrhosis	37.7% (26/69)	50% (16/32)	27.0% (10/37)	0.080
ALT (U/L)	53.2 ± 38.5	62.3 ± 42.6	45.3 ± 33.2	0.067
AST (U/L)	53.4 ± 31.9	60.1 ± 33.8	47.6 ± 29.4	0.104
Albumin (g/dL)	4.4 ± 0.3	4.3 ± 0.3	4.6 ± 0.3	0.002
Total bilirubin (mg/dL)	0.7 ± 0.4	0.7 ± 0.2	0.8 ± 0.4	0.143
Prolongation of prothrombin time (sec)	0.6 ± 0.7	0.7 ± 0.7	0.6 ± 0.7	0.436
Creatinine (mg/dL)	1.2 ± 1.2	1.5 ± 1.7	0.9 ± 0.4	0.086
α-fetoprotein (ng/mL)	425.9 ± 1219.4	589.6 ± 1469.6	284.2 ± 950.9	0.303
TNM stage (1:2:3)	22:36:11	7:14:11	15:22:0	< 0.001^a^
Follow up time (days)	928 ± 347	877.7 ± 373.7	970.8 ± 320.7	0.269
Mortality	10.1% (7/69)	18.8% (6/32)	2.7% (1/37)	0.044

Data are percentage or mean (standard deviation). Abbreviations: ALT, alanine aminotransferase; AST, aspartate aminotransferase; HBsAg, hepatitis B virus surface antigen; HCV, hepatitis C virus. A 2-tailed independent *t* test was used for continuous variables between recurrence and non-recurrence groups. Comparisons of nominal values were by Fisher's exact test except TNM stage.

a By a Pearson Chi square test.

### GLK is associated with HCC recurrence

**Figure 1:**
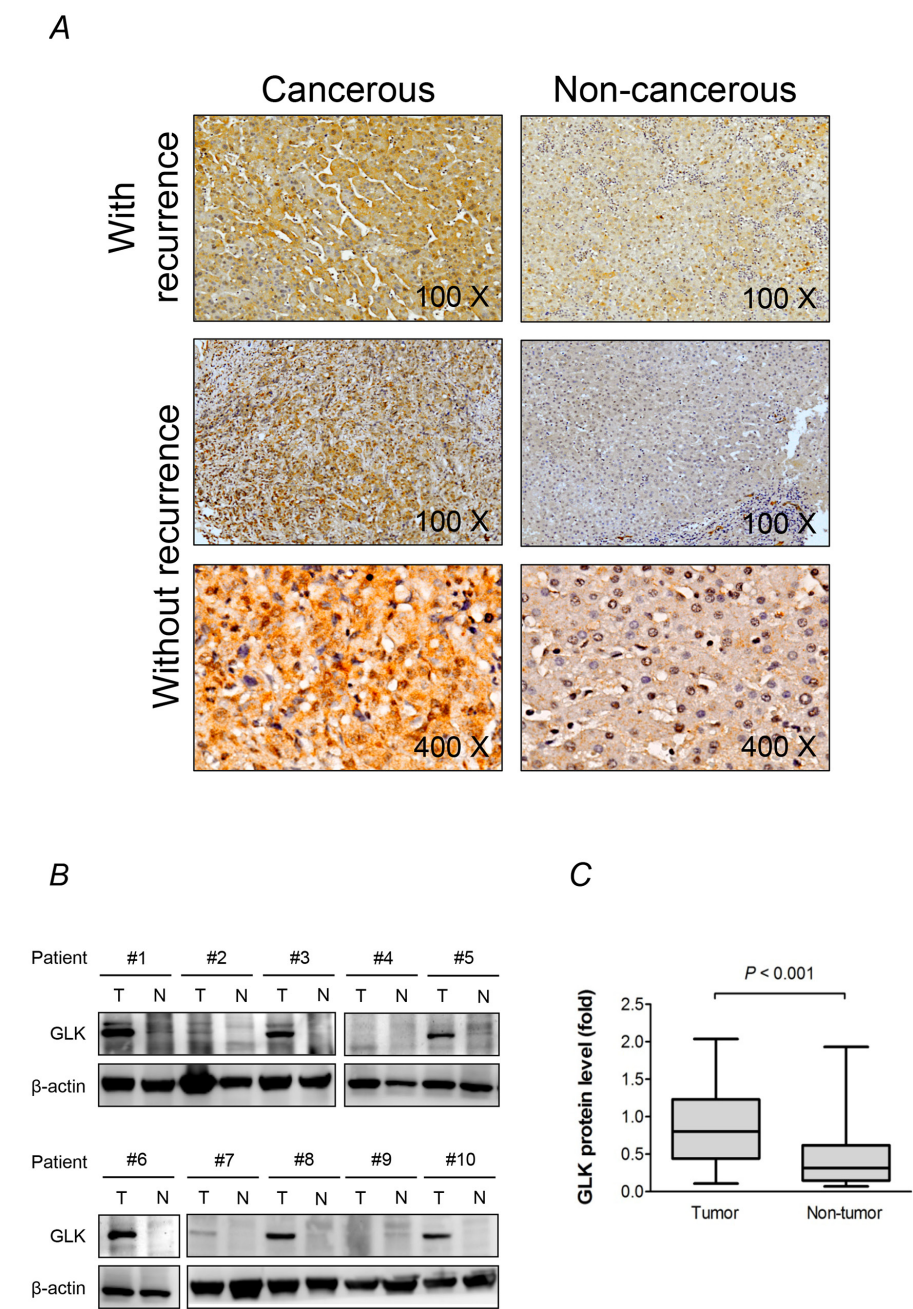
GLK is overexpressed in HCC **A.** Immunohistochemistry results from representative sections demonstrate overexpression of GLK in cancerous and adjacent non-cancerous paired liver tissue samples from patients with recurrent HCC. At higher magnification (× 400), the cellular distribution of GLK in hepatocytes is visualized. **B.** GLK in extracts generated from tumorigenic (T) and adjacent non-tumorigenic (N) tissue was detected by immunoblotting. **C.** Comparison of GLK protein levels from the immunoblotting results (n = 30) between cancerous and adjacent non-cancerous tissue shown as box-and-whisker plots (minimum, first quartile, median, third quartile, and maximum). Relative fold changes are normalized to β-actin. The *P-*value was obtained from the Mann-Whitney *U* test.

We evaluated the expression level and pattern of GLK in resected HCC tissue. Immunohistochemical analysis revealed that GLK expression was higher in cancerous tissue compared to adjacent non-cancerous tissue (Figure 1A). In addition, patients with recurrent HCC had similar GLK levels in cancerous liver tissue but higher GLK levels in non-cancerous tissue compared to patients who did not have recurrent HCC. Imaging at higher magnification indicated that GLK was predominantly expressed in the cytoplasm of hepatocytes. Western blot analyses of HCC tissue extracts confirmed that GLK was overexpressed in cancerous liver tissues (Figure 1B and 1C). Cancerous tissue generally had higher GLK proportion scores and higher Allred scores, but the GLK intensity scores were similar compared to adjacent non-cancerous tissue (Table 2). The proportion but not the intensity of GLK expression in non-cancerous liver tissues was associated with HCC recurrence.

**Table 2:** GLK expression in liver tissues of patients with HCC

GLK expression	Cancerous (n = 69)	Non-cancerous (n = 69)	*P*-value^1^	Cancerous		*P*-value^2^	Non-cancerous		*P*-value^3^
				Recurrence (n = 32)	Non-recurrence (n = 37)		Recurrence (n = 32)	Non-recurrence (n = 37)	
Intensity score (0-3)	1.0 ± 1.0	0.9 ± 0.8	0.187	1.1 ± 0.9	1.0 ± 1.0	0.448	1.1 ± 0.8	0.7 ± 0.9	0.057
Proportion score (0-5)	1.5 ± 1.4	1.1 ± 1.0	0.017	1.6 ± 1.2	1.5 ± 1.5	0.882	1.4 ± 1.0	0.8 ± 1.0	0.029
Allred score (0-8)	2.6 ± 2.2	1.9 ± 1.8	0.033	2.7 ± 1.9	2.5 ± 2.4	0.667	2.4 ± 1.6	1.5 ± 1.8	0.028

The intensity score is graded on scale from 0 to 3 (0 for no staining, 1 for weak staining, 2 for moderated staining, and 3 for strong staining). The proportion score is graded on a scale from 0-5 (0 for no staining, 1 for ≥ 1%, 2 for ≥ 10%, 3 for ≥ 33.3%, 4 for ≥ 66.7%, and 5 for 100%). Allred score (range 0-8) = intensity score + proportion score. Data are shown as mean ± standard deviation. *P*-values^1^ are from 2-tailed paired *t* tests. *P*-values^2^ and *P*-values^3^ are from 2-tailed independent *t* tests.

We next investigated factors associated with GLK expression in HCC tissue. Patients with different GLK proportion scores in cancerous tissue had similar degrees of necroinflammation and fibrosis. They also had similar incidences of HBV infection, HCV infection, steatosis, and liver cirrhosis (Supplementary Table 1). However, the GLK proportion score in non-cancerous liver tissue was correlated with the HCC recurrence rate. These results indicated that GLK was widely distributed in malignant liver tissues and in non-malignant liver cells of patients with HCC recurrence.

### GLK activates NFκB signaling in human hepatocytes

**Figure 2:**
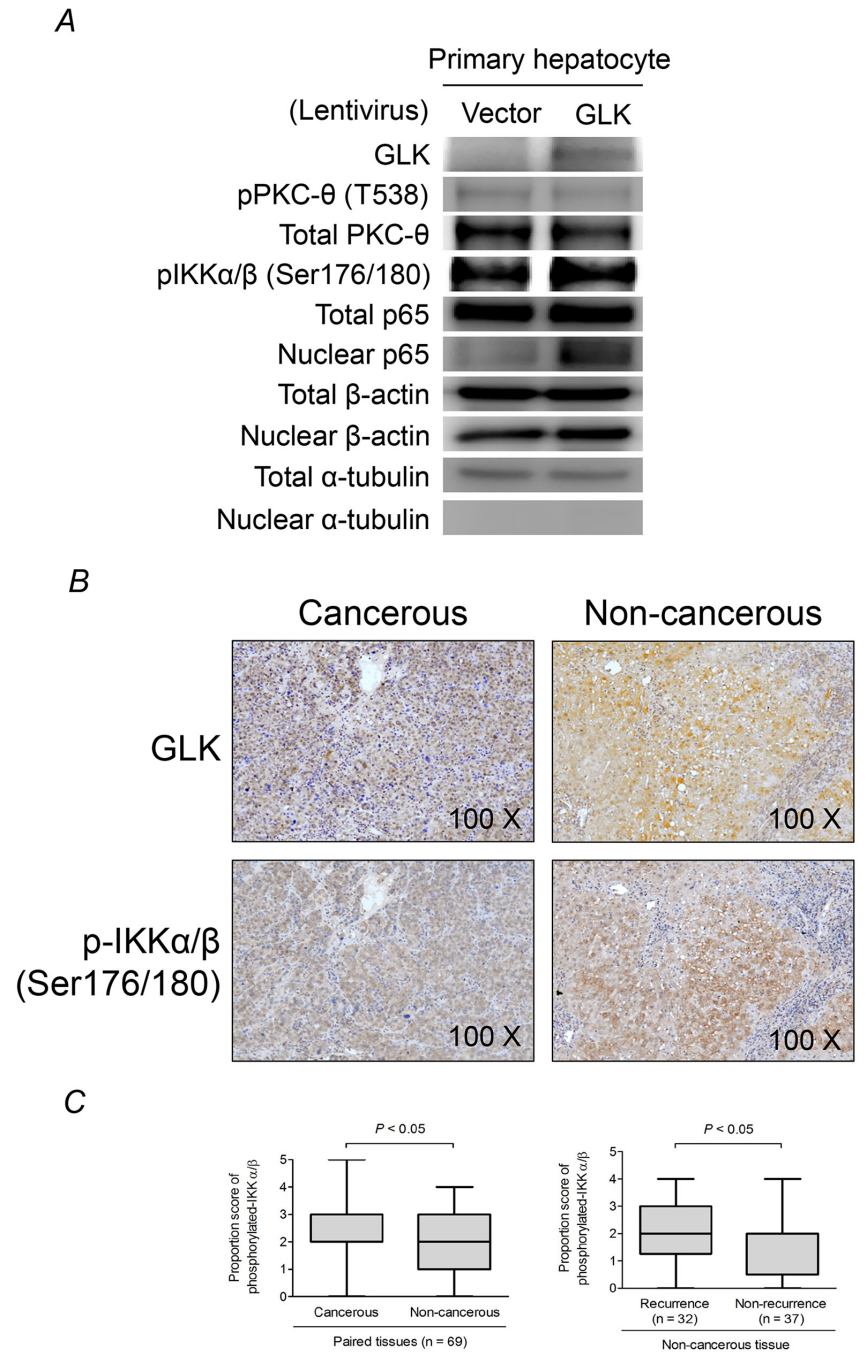
GLK induces PKC-θ-independent activation of NFκB **A.** Immunoblotting to detect PKC-θ phosphorylation and NFκB activation in primary human hepatocytes 48 hours after recombinant lentivirus infection. **B.** Detection of GLK and phosphorylated IKKα/β (Ser176/180) in cancerous and non-cancerous HCC tissue using immunohistochemistry. **C.** Comparisons of the proportion scores of phosphorylated IKKα/β between cancerous tissue and adjacent non-cancerous tissue (left), and non-cancerous liver tissue from patients with or without recurrent HCC (right), are shown as box-and-whisker plots (minimum, first quartile, median, third quartile, and maximum). The *P-*values in the left and right figures were obtained from Wilcoxon signed-rank and Mann-Whitney *U* tests, respectively.

We previously demonstrated that GLK induces PKC-θ phosphorylation at Thr538 and hyperactivation of NFκB, which is crucial for the progression of T helper (Th) 17 cell-mediated autoimmune disease [12]. Here, we examined whether GLK activated this signaling pathway in hepatocytes. GLK overexpression resulted in the activation of NFκB signaling as evidenced by increased IκB kinase (IKK) phosphorylation and p65 nuclear translocation but not PKC-θ phosphorylation in primary human hepatocytes (Figure 2A). GLK and phospho-IKK were observed in similar areas of serial sections of HCC tissue from equivalent biopsies (Figure 2B panel). Malignant tissue had higher phospho-IKK proportion scores compared to paired non-cancerous tissue samples from HCC patients, and showed a similar expression pattern to that of GLK in HCC tissue (Figure 2C, left). Furthermore, patients with recurrent HCC had higher IKK phosphorylation in non-cancerous tissue than patients who did not have recurrence (Figure 2C, right). Nevertheless, PKC-θ phosphorylation at Thr538 in HCC tissues was not correlated with GLK or IKK phosphorylation (Supplementary Figure S1A). Additionally, the levels of Th17-related cytokines including interleukin (IL)-1β, IL-6, IL-17, and tumor necrosis factor-α in serum were not correlated with the proportion or intensity of GLK (Supplementary Figure S1B). These results revealed that GLK-mediated NFκB activation in HCC was independent of the PKC-θ-Th17 signaling pathway.

### GLK enhances primary hepatocyte proliferation

**Figure 3:**
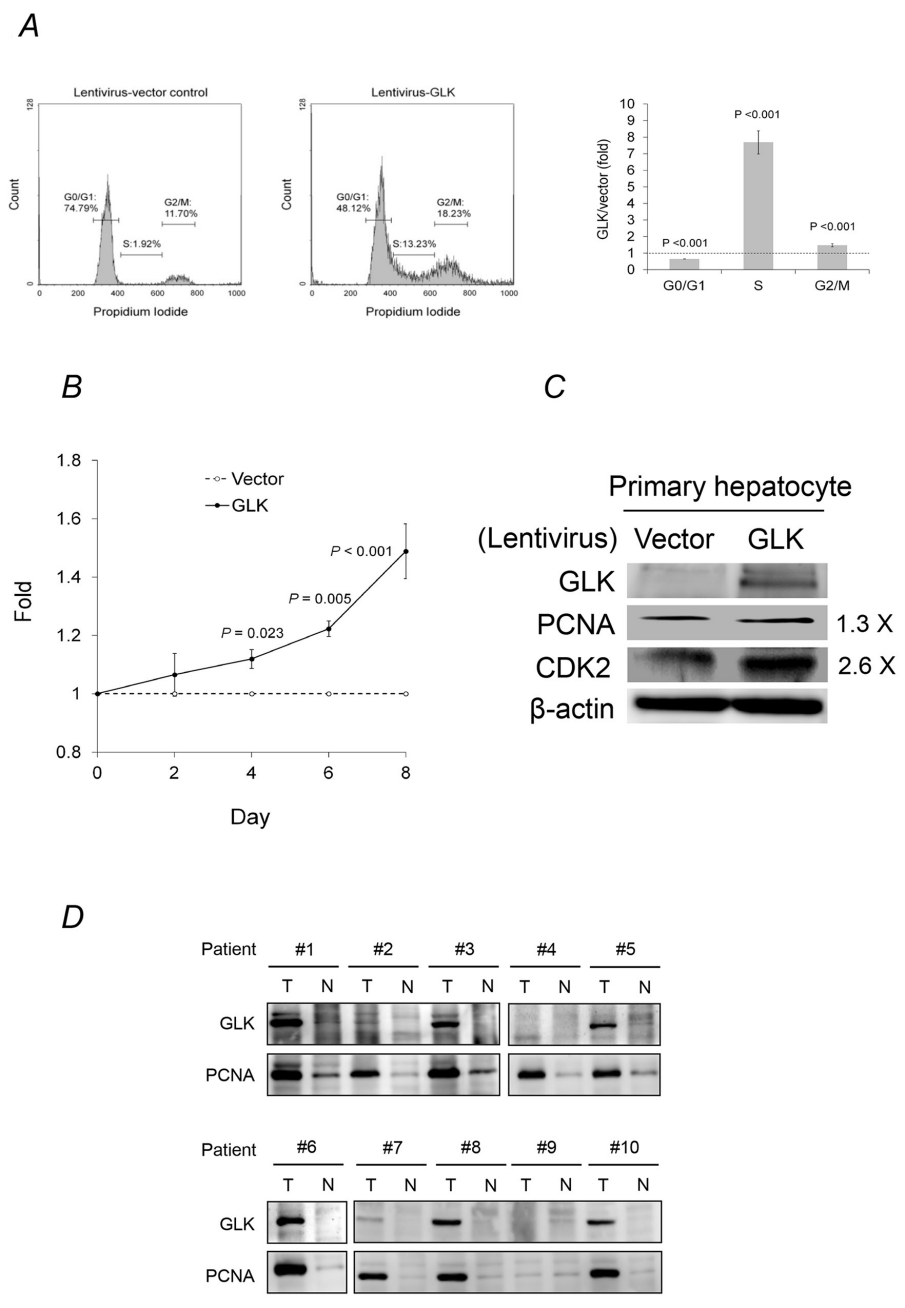
GLK modulates cell cycle progression in hepatocytes **A.** The cell cycle status of primary human hepatocytes with or without GLK overexpression was analyzed using propidium iodide staining and flow cytometry. **B.** Fold change in the proliferation of primary human hepatocytes with or without GLK overexpression was measured in triplicate and calculated at days 2, 4, 6, and 8. *P*-values were obtained from two-tailed independent *t*-tests. **C.** Levels of cell cycle markers in primary human hepatocytes detected using immunoblotting. **D.** Co-expression of GLK and PCNA in the same HCC tissue extracts as in Figure 1B. T, tumorigenic tissue; N, adjacent, non-tumorigenic tissue.

GLK stimulated cell cycle progression, which was reflected by a decrease in the percentage of cells in the G0/G1 phases and an increase in the percentage of cells in the S and G2/M phases, and accelerated primary human hepatocyte proliferation (Figure 3A and 3B). Proliferating cell nuclear antigen (PCNA) and cyclin-dependent kinase 2, two essential regulators of the G1/S phase, were increased by GLK in primary human hepatocytes. In addition, PCNA expression was correlated with GLK expression in HCC tissue (Figure 3D). These results indicated that GLK had a proliferative effect on human hepatocytes.

### The proportion of GLK in non-cancerous liver tissue predicts early HCC recurrence

**Figure 4:**
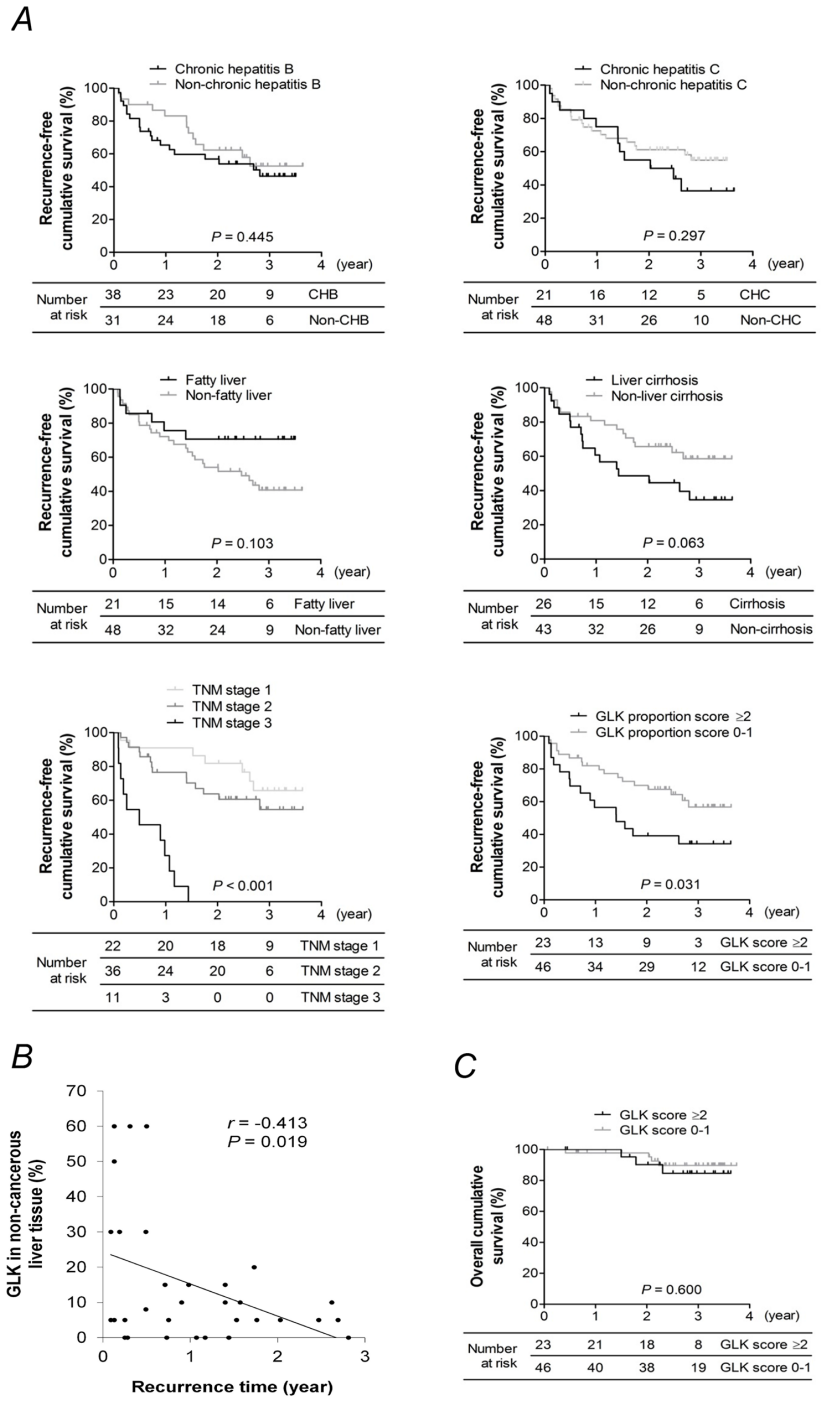
Kaplan-Meier analysis **A.** Recurrence-free survival rates for patients with or without chronic hepatitis B, chronic hepatitis C, liver cirrhosis, fatty liver, advanced TNM stage, or a GLK proportion score ≥ 2 in adjacent non-cancerous liver tissue. **B.** Correlation between the percentage of GLK expression in non-cancerous liver tissue and time of HCC recurrence. The coefficient *r* was obtained from Pearson's correlation test. **C.** Overall survival of patients with a GLK proportion score ≥ 2 or < 2 in adjacent non-cancerous liver tissue. *P-*values in (A) and (C) were obtained from log-rank tests.

Kaplan-Meier analyses revealed that patients who had a GLK proportion score ≥ 2 in non-cancerous liver tissue (Log-rank test, *P* = 0.031) or an advanced tumor stage (Log-rank test *P* < 0.001), but not with chronic HBV infection, chronic HCV infection, liver cirrhosis, or fatty liver, had a significantly lower recurrence-free survival rate than their counterparts (Figure 4A, panel). Multivariate Cox regression analyses showed that high GLK proportion scores in non-cancerous liver tissue as well as TNM stage were independent factors strongly linked to early HCC recurrence (Table 3). The percentage of GLK expression in non-cancerous liver tissue was inversely correlated (*r* = −0.413, *P* = 0.019) with recurrence time in 32 patients with recurrent HCC (Figure 4B). The overall survival rates in patients with different GLK proportion scores were similar (Figure 4C).

**Table 3:** Multivariate Cox regression analysis of HCC recurrence

Variable	Within 1 year		Within 2 years		Overall	
	Hazard ratio (95% CI)	*P*-value	Hazard ratio (95% CI)	*P*-value	Hazard ratio (95% CI)	*P*-value
Sex	0.05 (0.01 - 0.36)	0.003	0.22 (0.06 - 0.80)	0.021	0.61 (0.12 - 3.18)	.554
Age	1.05 (0.97 - 1.14)	0.208	1.03 (0.97 - 1.09)	0.348	1.03 (0.96 - 1.11)	.384
Alcohol	0.18 (0.01 - 2.83)	0.221	0.29 (0.06 - 1.54)	0.147	0.74 (0.06 - 8.51)	.809
Smoking	0.30 (0.03 - 4.32)	0.314	0.76 (0.20 - 2.81)	0.675	0.44 (0.05 - 3.85)	.462
Fatty liver	1.13 (0.30 - 4.32)	0.854	0.48 (0.18 - 1.29)	0.145	0.26 (0.06 - 1.21)	.087
HBV infection	13.16 (1.07 - 162.15)	0.044	2.55 (0.73 - 8.88)	0.142	2.41 (0.34 - 16.82)	.376
HCV infection	15.74 (1.08 - 229.90)	0.044	3.14 (0.74 - 13.26)	0.119	1.93 (0.23 - 15.86)	.541
Liver cirrhosis	0.84 (0.25 - 2.84)	0.783	1.20 (0.46 - 3.09)	0.711	3.93 (0.74 - 20.86)	.108
α-fetoprotein	1.00 (1.00 - 1.00)	0.632	1.00 (1.00 - 1.00)	0.671	1.00 (1.00 - 1.00)	.740
TNM stage	11.31 (3.13 - 40.94)	< 0.001	7.71 (3.17 - 18.74)	< 0.001	5.77 (1.77 - 18.82)	.004
GLK proportion score in non-cancerous tissue	2.59 (1.33 - 5.02)	0.005	2.18 (1.38 - 3.44)	< 0.001	3.06 (1.45 - 6.49)	.003
pIKK proportion score in non-cancerous tissue	0.98 (0.62 - 1.52)	0.913	1.24 (0.87 - 1.77)	0.233	2.22 (1.16 - 4.26)	.017

Abbreviations: CI, confidence interval; HBV, hepatitis B virus; HCC, hepatocellular carcinoma; HCV, hepatitis C virus; pIKK, phosphorylated inhibitor of nuclear factor kappa B kinase.

## DISCUSSION

We performed a comparative analysis of the GLK expression pattern (GLK signature) in cancerous and adjacent non-cancerous tissue in order to better understand HCC development and recurrence. We found that GLK was a marker of HCC, and that a broad distribution of GLK in non-malignant hepatocytes determined HCC recurrence. *In vitro* studies revealed that GLK had a proliferative effect on primary human hepatocytes. Thus, therapeutic strategies that target GLK may be effective for the treatment of HCC patients who have undergone hepatic resection.

GLK is a key regulator of T cell receptor signaling pathways and is critical for T cell functions. Indeed, GLK-knockout mice were shown to have impaired T cell proliferation, differentiation, and immune activation [12]. Upon T cell receptor signal transduction, GLK interacts with its upstream adaptor SLP-76, phosphorylates PKC-θ, and triggers downstream NFκB activation. Hyperactivation of this signaling cascade induces IL-17 production and contributes to the progression of autoimmune disorders such as systemic lupus erythematosus and adult-onset Still's disease [12, 15]. GLK also functions in other cell types. For example, it activates endophilin-mediated Jun-N-terminal kinase in human embryonic kidney 293 cells [16], mTOR signaling to modulate HeLa cell growth and viability [17], and post-transcriptionally regulates BH3-only proteins to regulate cell death in human osteosarcoma cells [11]. These data suggest that GLK is a crucial regulator of cell growth and death.

Here, we demonstrated that GLK promotes cell cycle progression and hepatocyte proliferation, which may contribute to hepatocarcinogenesis. Importantly, a GLK proportion score ≥ 2, which was indicative of GLK expression by more than 10% of non-tumorigenic hepatocytes, was linked to early HCC recurrence. This finding suggested that a small amount of GLK could have a strong oncogenic effect on hepatocytes, and that GLK was important for HCC recurrence. However, our assessment of GLK-related effects on the overall survival of HCC patients was limited by the low mortality rate and relatively short follow-up duration in this study. Furthermore, better management of HCC patients and medical interventions that prolonged the overall life-span of patients weakened the association between tumor recurrence and mortality. Therefore, similar survival rates were observed between patients with high and low GLK expression. Long-term follow-up studies are therefore required to assess the impact of GLK on HCC patient survival.

NFκB is critical for the cellular response to proinflammatory stimuli, and it links chronic inflammation to carcinogenesis [18–20]. NFκB can contribute to carcinogenesis [21–23] through its ability to regulate cell proliferation, apoptosis, immortalization, migration, and invasion [24–27]. It also has a critical role in the progression of hepatitis to HCC [28]. Most patients in our study had chronic viral hepatitis and 30% had been diagnosed with steatohepatitis. Persistent liver inflammation in these patients probably contributed to a robust NFκB activation. *In vitro* analyses demonstrated that cell cycle progression and increased PCNA were accompanied by GLK-dependent NFκB activation in hepatocytes. NFκB hyperactivity [29–31] and PCNA [32–34] have been shown to be essential for HCC recurrence. Therefore, a GLK-NFκB-cell proliferation axis may exist in the liver. PKC-θ is a pivotal mediator that connects GLK-NFκB signaling in T cells (with the exception of the liver) [12]. Moreover, the expression of Th17-related cytokine was not correlated with GLK expression. Therefore, GLK may bypass PKC-θ to stimulate NFκB activity in the liver.

An enhancement in kinase activity is a common phenomenon in various cancers. Sorafenib inhibits the activity of multiple tyrosine kinases. It is currently the standard of care for advanced HCC owing to its efficacy in prolonging overall survival and extending the duration of tumor progression in patients [35]. Unfortunately, a global phase III randomized, double-blind, placebo-controlled trial reported that adjuvant sorafenib after resection or ablation did not reduce the recurrence rate [36]. In this study, we demonstrated the clinical significance of GLK in HCC recurrence. The development of new therapeutics that target GLK would therefore improve HCC patient outcomes.

## MATERIALS AND METHODS

### Patients

This retrospective study was approved by the Institutional Review Board of the National Cheng Kung University Hospital (No B-ER-101-259). Resected liver tissue, clinical information, and laboratory data for 69 patients with HCC were obtained from the Tissue Bank and database at the Research Center of Clinical Medicine, National Cheng Kung University Hospital. All patients were negative for human immunodeficiency virus and autoimmune diseases.

### Antibodies

The rabbit anti-GLK antibody that was used for immunohistochemical analysis was purchased from LTK BioLaboratories (Taoyuan, Taiwan). Rabbit antibodies to detect GLK, protein kinase C (PKC)-θ, phosphorylated (Ser176/180)-IKKα/β, and the NFκB p65 subunit by western blotting were purchased from Cell Signaling Technology (Danvers, MA, USA). The rabbit anti-phospho (Threonine 538)-PKC-θ antibody was purchased from Thermo Fisher Scientific (Waltham, MA, USA). The rabbit anti-proliferating cell nuclear antigen (PCNA) antibody and horseradish peroxidase-conjugated goat anti-mouse and anti-rabbit antibodies were purchased from Abcam (Cambridge, UK). The rabbit anti-cyclin-dependent kinase (CDK) 2 antibody was purchased from Santa Cruz Biotechnology (Dallas, TX, USA). Finally, mouse antibodies to detect human β-actin and α-tubulin were purchased from Sigma-Aldrich (St. Louis, MO, USA).

### Lentivirus-mediated gene transfer

The lentiviral vector pReceiver-LV105 containing full-length GLK coding sequence (NM_003618.3) was purchased from GeneCopoeia (Rockville, MD, USA). The packaging plasmid pCMVdeltaR8.91 and VSV-G expression plasmid pMD.G were obtained from the National RNAi Core Facility, Academia Sinica, Taiwan. We co-transfected the GLK-lentiviral construct or control lentiviral vector with the pCMVdeltaR8.91 and pMD.G plasmids into 293T cells to generate recombinant lentiviruses. Culture medium was collected 48 hours post-transfection, sterilized using a syringe filter with a 0.22 μm pore size hydrophilic polyethersulfone membrane, and concentrated using ultracentrifugation. Primary human hepatocytes that were purchased and maintained in Hepatocyte Medium (ScienCell Research Laboratories, Carlsbad, CA, USA) were incubated with recombinant lentiviruses for 48 hours in the presence of 8 μg/mL polybrene (Sigma-Aldrich).

### Immunohistochemistry

Formalin fixed, paraffin-embedded tissue sections were de-paraffinized, rehydrated, and heated in 10 mM sodium (pH 6.0) for antigen retrieval. Hydroxy peroxide (3% in methanol) and blocking reagent were used to minimize endogenous peroxidase activity and non-specific signals. Tissue sections were then incubated with primary antibody at 4°C overnight. All slides were stained with 3,3′-diaminobenzidine substrate and counterstained with hematoxylin. The Allred score system was used to evaluate the staining results [13, 14]. The intensity score was graded on scale from 0 to 3 (0 for no staining, 1 for weak staining, 2 for moderated staining, and 3 for strong staining). The proportion score was graded on a scale from 0 to 5 (0 for no staining, 1 for ≥ 1%, 2 for ≥ 10%, 3 for ≥ 33.3%, 4 for ≥ 66.7%, and 5 for 100%). The Allred score (range 0 to 8) = intensity score + proportion score. Data were evaluated by a single experienced hepatopathologist who was blinded to the clinical data.

### Western blot analysis

Cancerous or adjacent non-cancerous liver tissue from 30 patients with HCC, or primary human hepatocytes, was lysed in RIPA buffer. Nuclear extracts were harvested using the Nuclear Extraction Kit (Chemicon International Inc., Billerica, MA, USA). Proteins were resolved on sodium dodecyl sulfate-polyacrylamide gel and electrotransferred onto polyvinylidene fluoride membranes. After blocking in 5% (w/v) bovine serum albumin or dry milk in Tris-buffered saline with 0.1% Tween 20, the membranes were incubated with primary antibody at 4°C overnight. After washing, the membranes were incubated with horseradish peroxidase-conjugated goat anti-mouse or anti-rabbit antibody. Signals were detected using a BioSpectrum Imaging System (UVP LLC, Upland, CA, USA). Full immunoblots are shown in Supplementary Figure S2.

### Cell cycle analysis

Following lentiviral transduction, primary human hepatocytes were fixed in 70% ethanol at −20°C overnight and treated with ribonuclease A (50 μg/mL) at 37°C for 30 minutes. The cells were then stained with propidium iodide (20 μg/mL) and then analyzed by flow cytometry (FACScan, Becton, Dickinson and Company, Franklin Lakes, NJ, USA).

### Enzyme-linked immunosorbent assays

Serum cytokines including interleukin (IL)-1β, IL-6, IL-17A, and tumor necrosis factor (TNF)-α were detected using the Ready-Set-Go ELISA kit (eBioscience, San Diego, CA, USA) according to the manufacturer's instructions.

### Statistical analysis

SPSS 17.0 for Windows was used for all statistical analyses. Continuous variables were compared using Student *t* tests or Mann-Whitney *U* tests for two independent groups, and paired *t* tests or Wilcoxon signed-rank tests for two related groups. Nominal variables were compared using Fisher's exact tests or Pearson Chi square tests. Multivariate Cox regression analyses were performed to evaluate factors associated with HCC recurrence. Kaplan-Meier analyses and log-rank tests were used to assess the significance of various clinical features on recurrence-free survival or overall survival. Significance was defined as *P* < 0.05, and all *P*-values were two-tailed.

## SUPPLEMENTARY MATERIALS FIGURES AND TABLES


