# Supplementary material for: Prediction of early hepatocellular carcinoma recurrence using germinal center kinase-like kinase
Source: Oncotarget. 2016 Jun 20;7(31):49765–76. doi: 10.18632/oncotarget.10176 (PMC5226546; doi:10.18632/oncotarget.10176)
Supplement: Supplementary file 1 [file oncotarget-07-49765-s001.pdf]

Prediction of early hepatocellular carcinoma recurrence using germinal center kinase-like kinase

SUPPLEMENTARY FIGURES AND TABLE

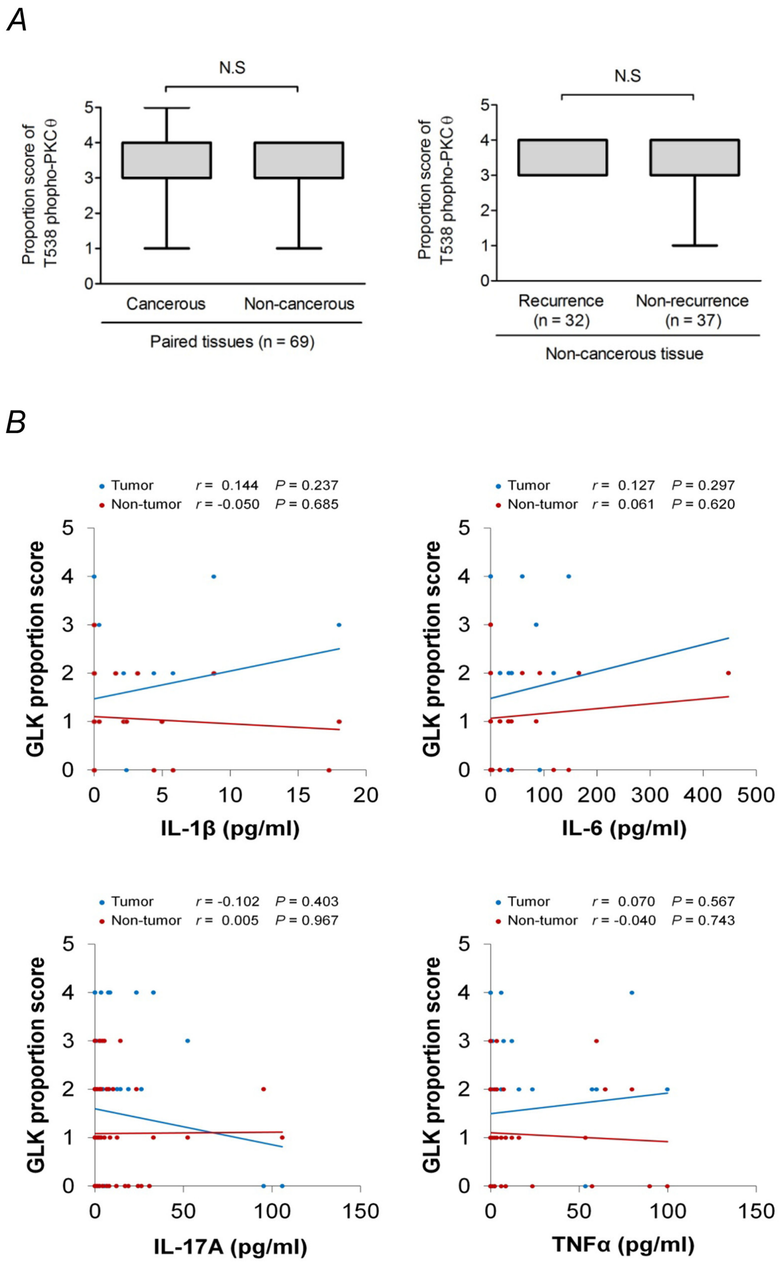

Supplementary Figure S1: A. Comparisons of the proportion scores of phosphorylated PKC-θ (Thr538) from the immunohistochemistry results between cancerous and adjacent non-cancerous tissue, and in non-cancerous liver tissue between patients with and without recurrent HCC, are shown as box-and-whisker plots (minimum, first quartile, median, third quartile, and maximum). The *P*-values in the left and right figures were obtained from Wilcoxon signed-rank and Mann-Whitney *U* tests, respectively. N.S., not significant. **B.** No correlation between the GLK proportion score and levels of Th17-related cytokines in serum was observed. The coefficient *r* was obtained from Pearson's correlation test.

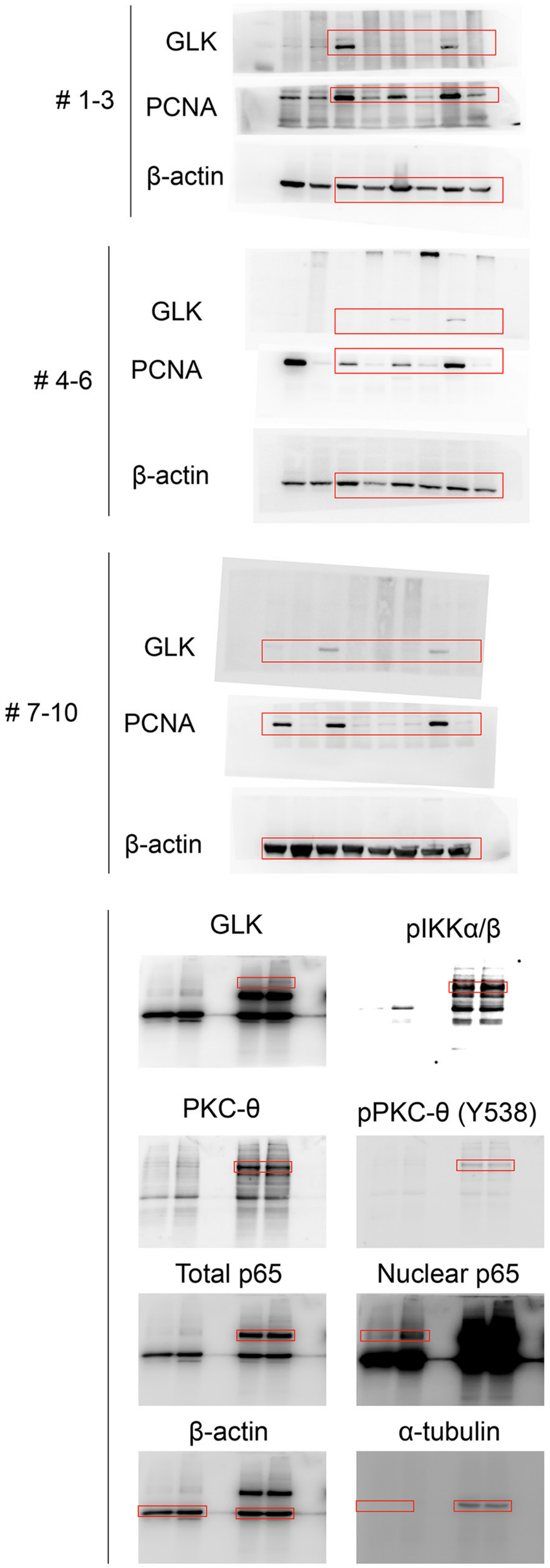

Supplementary Figure S2: Full immunoblots with indicated areas of selection.

Supplementary Table S1: Comparisons of characteristics of patients with different GLK proportion scores in HCC liver tissue

Variable	Cancerous tissue		*P*-value	Non-cancerous tissue		*P*-value
	Score 0-1 (n = 32)	Score ≥2 (n = 37)		Score 0-1 (n = 46)	Score ≥2 (n = 23)	
Sex (M:F)	24:8	27:10	1.000	33:13	18:5	0.772
Age (years)	60.7 ± 12.4	59.8 ± 9.6	0.742	60.4 ± 10.4	59.8 ± 12.0	0.819
Alcohol	18.8% (6/32)	18.9% (7/37)	1.000	15.2% (7/46)	26.1% (6/23)	0.334
Smoking	25.0% (8/32)	18.9% (7/37)	0.572	19.6% (9/46)	26.1% (6/23)	0.550
Fatty liver	34.4% (11/32)	27.0% (10/37)	0.603	34.8% (16/46)	21.7% (5/23)	0.406
HBV or HCV infection	78.1% (25/32)	86.5% (32/37)	0.526	84.8% (39/46)	78.3% (18/23)	0.517
Knodell inflammation score	3.6 ± 1.9	3.9 ± 2.2	0.604	3.8 ± 2.3	3.7 ± 1.9	0.801
Ishak fibrosis score	4.2 ± 1.6	3.7 ± 1.8	0.229	3.9 ± 1.7	4.0 ± 1.7	0.692
Liver cirrhosis	31.3% (10/32)	43.2% (16/37)	0.331	39.1% (18/46)	34.8% (8/23)	0.796
TNM stage (1:2:3)	13:13:6	9:23:5	0.197[a]	14:26:6	8:10:5	0.517[a]
Recurrence	43.8% (14/32)	48.6% (18/37)	0.810	37.0% (17/46)	65.2% (15/23)	0.040
Mortality	9.3% (3/32)	10.8% (4/37)	1.000	8.7% (4/46)	13.0% (3/23)	0.679

Data are percentage or mean (standard deviation). The proportion score is graded on a scale from 0-5 (0 for no staining, 1 for ≥ 1%, 2 for ≥ 10%, 3 for ≥ 33.3%, 4 for ≥ 66.7%, and 5 for 100%). Abbreviations: ALT, alanine aminotransferase; AST, aspartate aminotransferase; HBV, hepatitis B virus; HCC, hepatocellular carcinoma; HCV, hepatitis C virus. A 2-tailed independent *t* test was used for continuous variables. Comparisons of nominal values were by Fisher's exact test except TNM stage. [a]By a Pearson Chi square test.
